# Eucalyptus as a Disinfectant, Antiseptic and Epidemic Remedy

**Published:** 1877-01-20

**Authors:** A. M. Woodward

**Affiliations:** Tunkhannock, Pa.


### EUCALYPTUS AS A DISINFECTANT, ANTISEPTC AND EPIDEMIC REMEDY.

---

By A. M. Woodward, M.D., Tunkhannock, Pa.

---

I propose giving you a little of my experience in the use of Eucalyptus. About the last of August, 1875, I had a case of intermittent fever which quinine did not touch, from the fact that it would not stay in the stomach so long as while it was going down, even at the intervals between the sweating stage and chill. I suppose you will say that I did not prepare the stomach for the quinine. The Eucalyptus prepared the stomach by antidoting the poison that produced the trouble, and the case was cured in three days.—no more chills after the first fifteen drops of Eucalyptus were administered. It proved to be the only remedy the stomach would recognize as *the* tool which nature or the vital force required to work with in the case, and the beauty of it in such cases is, you can give it with all certainty without regard to stage, or, as the old folks used to say, "idiosyncrasy." You can call it anti-periodical in this case, or what you please. I only pretend to say, it was the specific antidote or remedy for the case.

Case 2.—"Facial neuralgia." Lady, aged 55, bilious and sanguine temperament; had been troubled by spells for several years. All the usual remedies failed to even afford relief. From its periodic character I tried Eucalyptus. (I should have said that the patient possessed an erysipelatous diathesis.) From the first fifteen-drop dose, I could see an amendment, and in twenty-four hours the neuralgia was gone; and at the usual time for it to return this fall, it did not put in an appearance. There were slight symptoms, but two or three doses of Eucalyptus were sufficient to set all right again.

Case 3.—*As an Antiseptic and Disinfectant.* Having a case of retained placenta at the fourth month, and not being called until the odor in the room became almost unbearable, I removed what I could of the putrid placenta, and ordered vaginal enemas of tepid water, adding a little castile soap, and used on my hands carbolic and salicylic acid, without much effect. I then tried Eucalyptus, which was at once the sufficient disinfectant. I at once returned and ordered the vaginal enemas changed to Eucalyptus put in alcohol equal parts, adding a teaspoonful to one-half pint or a pint of tepid water, and use at once, the enemas being repeated every three hours. Suffice it to say, the next day when I called there was no putrescency about the room or bed, although there were pieces of the placenta passed at several different times after the Eucalyptus was commenced, and the patient made a good recovery.

CASE 4.—Lady, aged 58. General erysipelas, with putrid dysenteric passages. Case pronounced hopeless by others. I gave a dose of Eucalyptus (15 drops), followed by veratrum gtt. vii, water oz. iv, a teaspoonful to be given every half hour until a moisture appeared on the skin. The passages spoken of occurring every few minutes before taking the Eucalyptus, did not occur but once after the first fifteen drops in twelve hours, and no more putrescency. After the first dose I prepared fluid extract Eucalyptus dr. ij, water oz. iv. Dose, a teaspoonful alternately with the veratrum. The patient made a good recovery.

CASE 5.—Child aged 8 years. Diphtheria. Had been treated three days "regular," and one case in same family treated "regular," died of same disease. Found the child's mouth black as ink; diphtheria coating as thick as one-half the length of the uvula; pulse asthenic, and breath very putrid. This was the only child left in the family, and I considered the responsibility, the parents being willing to risk the change of doctors. I made a gargle of tincture Baptisia dr. j, Eucalyptus dr. iss, water oz. iv, to be used, a teaspoonful as a gargle every hour, and one-half teaspoonful taken alternately with the gargling. Also, tinc. veratrum gtt. v, aconite gtt. vij, water oz. iv. M. S. One teaspoonful every hour. Sharp vinegar applied to the throat externally. Was to call again next day, but must confess I did not care about getting quite there until I heard the child was alive, and to my astonishment I found the mouth and throat as clean and natural in color as I could wish; tonsils somewhat swollen, but of natural color; pulse full and normal. Result, a good recovery.—*Eclectic Med. Jour.*

